# Influence of computers in students’ academic achievement

**DOI:** 10.1016/j.heliyon.2022.e09004

**Published:** 2022-02-24

**Authors:** Sofia Simões, Tiago Oliveira, Catarina Nunes

**Affiliations:** NOVA Information Management School (NOVA IMS), Universidade Nova de Lisboa, Campus de Campolide, 1070-312, Lisboa, Portugal

**Keywords:** Academic achievement, Computers, Family, Learning, Students

## Abstract

With fast-growing technology, schools have to adapt and use technology constantly as a tool to grow. This study aims to understand the influence of computer factors on students' academic achievement. We propose a model on the influence of computer attitudes, computer learning environments, computer learning motivations, computer confidence, computer use, computer self-efficacy, loneliness, mothers' education, parents' marital status and family size on academic achievement (AA). To validate the conceptual model, 286 students aged 16–18 years old answered an online questionnaire. The most important drivers that positively affect AA are computer use, employment motivations, and mothers' education. While enjoyment attitudes, school environment, interest motivations, and loneliness influence AA negatively. Also, family size and computer self-efficacy work as moderators, and computer use works as a mediator between computer learning environments and academic achievement.

## 1 Introduction

Countries are constantly facing everchanging economic challenges and social transformations due to globalisation and technology development. Education helps overcome these challenges by developing knowledge and high skills, allowing better opportunities and faster economic progression (OECD, 2019). Computers and information technology have become key to educational institutions worldwide (Hsu and Huang, 2006). With the advantages of the digital era through digital markets, advanced scientific and social networks, there is a growth in innovation, development and employment (OECD, 2015). Education needs to adapt to social changes, students' needs, and technology growth (OECD, 2019), the perfect example of this adaptation is during the recent pandemic. The COVID-19 pandemic (meaning "CO" - corona; "VI" – virus; "D" – disease; "19" - "2019″) started in December 2019 in Wuhan, a province of China. It is caused by a highly contagious virus that has already claimed millions of lives worldwide (Roy et al., 2020). The virus forced schools to close, and since classes had to continue, teachers and students had to adapt, resorting to virtual classes (Ng and Peggy, 2020). However, it impacted academic life in yet unknown dimensions (Rajkumar, 2020).

Digital technology provides access to high-quality learning and consequently allows schools to develop their teaching and learning methods (Ertmer et al., 2012). Nonetheless, access to computers at home or the internet is not equal in every dwelling, and some students have the disadvantage of not having parental support or engagement to learn by themselves online. For these reasons, the pandemic can bestow tremendous advantages in digital education and academic achievement or significant disadvantages, mostly in developing countries. Therefore, access to technology is not enough; fostering a close relationship between families and teachers is essential (OECD, 2020). Technology has been an invaluable tool, and it is being taken under consideration in students' academic achievement, including not only in access to the internet but also the way students use it (Levine and Donitsa-Schmidt, 1998; Torres-Díaz et al., 2016; Voogt et al., 2013). Schools are expected to have a particular concern regarding integrating computers in classroom teaching (Schmid and Petko, 2019), and technical devices such as computers, laptops, tablets and mobile phones should be included wisely in adolescent education. Through the information gathered, this study was motivated mainly by the atual pandemic context and the important role technology has on the academic achievement.

Over the years, researchers have tried to identify the variables that contribute to academic excellence in an attempt to understand which factors lead to better students' performance (Valli Jayanthi et al., 2014). A vast number of studies have been conducted to identify predictors of academic achievement (Gonzalez-pienda et al., 2002; J. Lee, Shute and Lee, 2010; Suárez-álvarez et al., 2014) although few have studied computer influences on the prediction of students' academic achievement.

Since there is a need to extend innovations in education (Admiraal et al., 2017), we identified a need to investigate how students' relationships with computers impact their academic performance to understand the real impact of computers on schooling. To the best of our knowledge, some studies address computers' impact on academic achievement, but the data available is not totally enlightening. With the actual context of the pandemic, this subject gains additional importance, comparing technology use and academic achievement (AA) in such a tumultuous time for the world. This study presents three contributions. **Firstly**, it identifies which the best computer-related determinants to understand AA are through a research model that combines computer-related variables to students' grades. In this way, we identify the factors that lead to better academic achievement, helping schools and parents use them as a strategic advantage. **Secondly**, it investigates the moderation effect of family size and computer self-efficacy and the mediation effect of computer use between the factors identified and AA. **Finally**, to understand how the COVID-19 pandemic is influencing students' AA, using the variable loneliness, we explore how forced social isolation affected the use of computers and students' academic achievement in the pandemic period.

A literature review is presented in the next section. Section 3 introduces a theoretical model explaining academic achievement. Section 4 elucidates on the data-collection methods, followed by the results in Section 5. The results are discussed in Section 6, and conclusions are outlined in the final section.

## 2 Literature review and hypotheses

### 2.1 Computer attitudes

Attitudes and perceptions play a pivotal role in learning behaviours. Some researchers tested a model based on the concept of the attitude-behaviour theory, which argues that beliefs lead to attitudes, and attitudes are an essential factor to predict behaviour (Levine and Donitsa-Schmidt, 1998). They predicted that computer use leads to more computer confidence and positive attitudes towards computers, and these elements influence each other. The computer attitudes refer to the opinion of students about: the stereotypes of those who use the computer the most – stereotypes; the use of computers for education purposes – educational; and about the use of the computer for fun – enjoyment. In their view, student achievement is a reflection of their behaviour in school. Even with the change of technology over time, recent studies support their theory that positive computer attitudes and positive computer confidence continue to lead to better outcomes (Lee et al., 2019). **Stereotypes** associated with computers are usually on gender, proving the idea that women have less computer knowledge than men (Punter et al., 2017). However, there are no results on how other stereotypes, such as the lack of computer use by athletes', or even if the concept of people who use computers are considered nerds, negatively affects the confidence of those who use computers.

Regarding the attitudes of **enjoyment and educational** use of computers, there is no consensus in the literature. Some researchers found a positive association between students' academic achievement and computer use for interactive social media and video gaming, as well as for educational purposes (Bowers and Berland, 2013; Tang and Patrick, 2018), although other researchers have found that students who play more videogames have worse results in school (Bae and Wickrama, 2015), some previous studies suggest that the technology intervention has a positive effect on students' attitudes toward the use of computers for educational purposes (Gibson et al., 2014). Others show concerns on the effects of technology and social media use on students' outcomes and confirm that students who have lower grades spend more time using computers for fun (Bae and Wickrama, 2015; Tang and Patrick, 2018), others find no evidence that using computers for fun causes higher or lower achievement (Hamiyet, 2015). Milani et al. (2019) demonstrated that using computers with moderate levels of video gaming may improve student achievement because it increases visual-spatial skills (Milani et al., 2019) when complemented with educational use such as homework, extracurricular activities, and reading (Bowers and Berland, 2013). Regarding the effect on computer confidence, we expect students to feel confident about using computers when using them for school (Claro et al., 2012) and even more when using them for recreational purposes. Taking this background into account, we propose the following hypotheses.

H1a Educational attitudes have a positive effect on computer confidence.

H1b Educational attitudes have a positive effect on academic achievement.

H2 Stereotype attitudes have a negative effect on computer confidence.

H3a Enjoyment attitudes have a positive effect on computer confidence.

H3b Enjoyment attitudes have a negative effect on academic achievement.

### 2.2 Learning environments and motivations

The environment where students learn can affect their attitudes (Hsu and Huang, 2006). Studies have found that students achieve higher grades when they have a computer at **home** (Fairlie, 2012; Fairlie et al., 2010) and use it daily to facilitate their school work (Gu and Xu, 2019), suggesting that home computers improve educational outcomes and computer skills, leading to more efficient use of computers (Fairlie and London, 2012). Many researchers pointed to a positive impact of computer use in **schools** on students' educational outcomes (Bayrak and Bayram, 2010; Murillo-Zamorano et al., 2019; Xiao and Sun, 2021). The integration of computers in the classroom positively influences the interaction between students and increases learning and teaching (Murillo-Zamorano et al., 2019). Experimental class manipulations using a computer in class were tested over the years, with positive results: students' academic achievement increases when a computer assists them in learning (Bayrak and Bayram, 2010). However, most students show dissatisfaction with the learning environment of schools (Hsu and Huang, 2006). So, we propose that home and school environments positively influence computer use in general and student achievement particularly, as hypothesised below.

H4a Home environments have a positive effect on computer use.

H4b Home environments have a positive effect on academic achievement.

H4c Computer use mediates the effect of home environment on academic achievement

H5a School environments have a positive effect on computer use.

H5b School environments have a positive effect on academic achievement

H5c Computer use mediates the effect of school environment on academic achievement

Regarding motivations, several types of motivations have already been studied to predict academic achievement, and the best predictor so far is associated with interest. If the student is interested, he will engage in the activity independently, and there is also evidence that **interest motivations** directly affect reading achievements (Habók et al., 2020). When analysing students' motivations for using computers, studies show that using computers at school and for schoolwork results in higher motivation when studying and positively impacts academic achievement (Partovi and Razavi, 2019). Likewise, when the students' perceptions of learning motivations are improved, there is an increasing computer use by the students and, as a result, it enhances their computer self-efficacy - perceived skill on the use (Rohatgi et al., 2016) - indirectly (Hsu and Huang, 2006). Therefore, in order to increase computer self-efficacy, students need to use computers more frequently. Previous results indicate that interest motivations positively affect computer use and computer self-efficacy, predicting that when student interests in computers are higher, student computer self-efficacy increases. Students are also motivated by **employment** and recognise that computer abilities can help them get a good job (Hsu and Huang, 2006). This factor can be predicted by self-efficacy because it defines the confidence and ability on achieving success (Serge et al., 2018). A study showed that learners who are more engaged and motivated use more technology for their learning purposes, most likely for individual learning than for collaborative tasks (Lee et al., 2019). Regarding the use of technology, students who use it more are more motivated to do it and have better grades (Higgins, Huscroft-D’Angelo and Crawford, 2019), and students who are motivated by attaining better grades tend to use e-learning more (Dunn and Kennedy, 2019). In line with the literature, we expect the confirmation of the presented hypotheses.

H6a Interest motivations have a positive effect on computer use.

H6b Interest motivations have a positive effect on academic achievement.

H6c Interest motivations have a positive effect on computer self-efficacy.

H7a Employment motivations have a positive effect on computer self-efficacy.

H7b Employment motivations have a positive effect on academic achievement.

### 2.3 Computer confidence, computer use & computer self-efficacy

Hands-on experience with technology is the most important factor in increasing students' **confidence** while using it and consequently increasing their perceived **computer self-efficacy** (Hatlevik and Bjarnø, 2021). Students with access to a computer are more involved and interested in their classwork (Gibson et al., 2014). Higher commitment to school, curiosity, and positivism can help students develop motivation and interest in school subjects, leading to higher self-efficacy and consequently better academic achievement (Stajkovic et al., 2018).

H8 Computer use has a positive effect on computer confidence.

H9 Computer confidence has a positive effect on computer self-efficacy.

H10 Computer confidence has a positive effect on academic achievement.

H11 Computer use has a positive effect on academic achievement.

We know from previous literature that employment motivations positively influence academic achievement, and computer self-efficacy is also a significant influence factor on employment (Serge et al., 2018) to explain academic achievement, so we believe that computer self-efficacy can moderate this relation by proposing H14.

H12 Computer self-efficacy moderates the effect of employment motivations on academic achievement.

### 2.4 Loneliness

Due to the coronavirus pandemic, schools were closed to slow down the virus transmission as a control measure, affecting half of the students globally (Viner et al., 2020). Schools were forced to adapt during coronavirus outbreaks since campus classes were suspended, and online platforms have been exploited to conduct virtual classes (Ng and Peggy, 2020). Ng and Peggy (2020) states that virtual classes can improve students' learning outcomes if all students are self-disciplined. However, self-isolation may affect people's mental health (Roy et al., 2020), primarily impacting adolescents, influencing their behaviours and achievement in academic pursuits. Interaction with others is a pivotal factor for academic performance since students who engage with colleagues and teachers tend to have more academic success than those who study by themselves (Torres-Díaz et al., 2016). **Loneliness** or social isolation is linked to anxiety and self-esteem (Helm et al., 2020), leading to unhealthy smartphone use (Shen and Wang, 2019) and sedentary behaviours (Werneck et al., 2019), motivating us to posit the following.

H13 Loneliness has a negative effect on academic achievement.

### 2.5 Family and students' factors

Technology use is linked to additional factors that influence adolescents' academic outcomes such as family socioeconomic factors – in particular, parents' occupation, marital status (Abosede and Akintola, 2016; Asendorpf and Conner, 2012), parents' educational level (Chesters and Daly, 2017) and family size - and student socio-emotional factors - such as relationship with colleagues, student motivation and anxiety (Balogun et al., 2017). Family involvement and closeness to younger progeny have positive impacts on their achievements (Fang, 2020), so we believe that the relation between using computers in a school environment on academic achievement, verified above, may change depending on the family size. Also, we know from the previous results that computer use has increased with the pandemic due to online classes, and family context has a significant impact on home computer use, so we predict a moderation effect on the relation between computer use and academic achievement. The psychological status of parents, mostly their marital status and economic status, has a powerful association with the family environment and consequently on their child's educational attainments (Poon, 2020). We predict there is a positive impact of mothers' education on academic achievement since the maternal figure is the most relevant for children (Abosede and Akintola, 2016). Expecting that the higher the level of education of mothers, the better the students result at school, also, we predict that parents being married have a positive influence on students' results, H15 and H16.

H14a Family size moderates the school environment on academic achievement.

H14b Family size moderates computer use on academic achievement.

H15 Parents marital status has a positive effect on academic achievement.

H16 Mothers' education has a positive effect on academic achievement.

According to their age and gender, students' grades can differ independently of their family characteristics: female students tend to achieve higher scores than male students (Valli Jayanthi et al., 2014) and older students showed lower grades compared to younger students (Chowa et al., 2015). Some of these factors are not of primary interest for this study. Nevertheless, it is crucial to include them in the research to control for bias since they influence the association between the use of technology and adolescents' outcomes (Tang and Patrick, 2018). We have therefore used age and gender as a control variable on our research model.

### 2.6 Conceptual model

**Figure 1:**
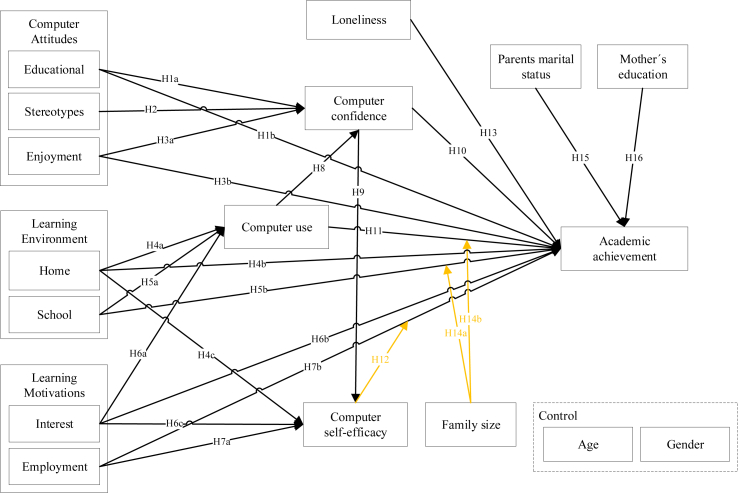
Conceptual model.

Figure 1 illustrates our proposed model. We focus our research on computers and their influence on academic achievement. The drivers shown in the research model emerged from the literature above. We first gathered information and identified the main factors that influence academic achievement through computer use, and from the most significant constructs relating to computers and academic achievement, we examined and analysed their viability on the study. From the computers' context, the most significant constructs found were computer attitudes (educational attitudes, enjoyable attitudes, stereotypes attitudes), computer use, computer confidence (Levine and Donitsa-Schmidt, 1998), computer self-efficacy, learning environments (home environment, school environment) and learning motivations (interest motivations, employment motivations) (Hsu and Huang, 2006). We identified loneliness as the most relevant construct from the pandemic context considering its impact on academic achievement (Helm et al., 2020). We identified mothers' education, marital status, and family size as the most relevant influencers from the family context. Finally, with our central construct, academic achievement, we are trying to understand how it is impacted by computers, the pandemic and family factors from students' points of view. So, the proposed model tries to predict AA through students' computer attitudes, learning environments, learning motivations, computer confidence, computer use, computer self-efficacy and loneliness, adding sociodemographic data related to students and their families - parents' marital status, mothers' education and family size, where the latter only works as a moderator, including two additional control variables, age and gender. This model integrates several constructs on the literature relevant to the study of computers influence on academic achievement since is essential to fortify and unify the knowledge in this investigation field. As explained above, the model merges two existing models (Hsu and Huang, 2006; Levine and Donitsa-Schmidt, 1998), allowing us to update the previous results and test new hypothesis. Additionally, the integration of the covid pandemic context brings a different and important analysis of today's reality.

## 3 Methods

### 3.1 Participants and procedure

For this study, we developed a questionnaire for students enrolled in public high schools. The survey, with an estimated completion time of 8 min was sent by e-mail to several schools in Portugal to achieve more diversity within the collected answers. The participants consented to the use of their information as long as it was anonymous and confidential. The questionnaire was answered online and comprised 26 closed questions (please, see Appendix A) inquiring about computer attitudes, motivations, use at home and school, frequency of use, students' grade average from 0 to 20 marks, and sociodemographic information. With this data, we can compare and analyse the impact of their type of use and opinion about computers on their achievement in school. The study's target population were 16 to 18-year-old adolescents in the 10^th^, 11^th^ and 12^th^ grades at secondary schools. This range of students allowed us to surround a group of people with similar maturity and identical needs in digital use. We chose to study public school students because teaching methods in private schools are quite different, as are the type of students and families who choose private schools. Also, most students in Portugal study at public schools, and it seems more coherent to study only public education since it is more accessible to address. According to the Ethics Committee of NOVA IMS and MagIC Research Center regulations, this project was considered to meet the requirements, being considered approved.

### 3.2 Data

A pilot test with 30 answers allowed us to comprehend the viability of some survey questions and their order, and afterwards, when evaluating the model, the strength of constructs led us to drop a few items due to the lack of importance and correlations within them. The pilot test allowed us to improve the questionnaire to facilitate answering and adapt the research model initially built. After the complete collection of data, we considered only student responses 100% completed, amounting to 286 valid responses, from a total of 465 answers. We had 98 boys and 188 girls among the respondents, with an average age of 17 years old, with an average global grade of 15 points (on a scale from 0 to 20). Students' academic achievement was measured through students' average grades - on reading, mathematics and global average grade. Computer use was measured through a scale range from 1 (never) to 5 (every day) to measure the frequency of use. A 3-item loneliness scale was used to assess the loneliness construct (Hughes et al., 2004) based on the UCLA Loneliness Scale (Russel, 1996). This scale has been used in several studies recently (Helm et al., 2020; Liu et al., 2020; Shen and Wang, 2019) to study loneliness as a consequence of the coronavirus. The remaining items, apart from the demographic variables (age, gender, marital status, mothers' education, family size), were measured through a scale range from 1 (strongly disagree) to 5 (strongly agree).

## 4 Analysis and results

We used structural equation modelling (SEM) to test the relations estimated in our theoretical model and its effects (Marsh et al., 2004). Consequently, we applied partial least squares (PLS), a method used to develop theories in explanatory research. The use of PLS-SEM is to maximise the explained variance in the dependent constructs and evaluate data quality, knowing that it is a method that works better on bigger sample sizes and larger complexity with less restrictive assumptions on data (Joe F Hair et al., 2014). We used the partial least squares method as the recommended two-step approach that first tests the reliability and validity of the measurement model and then assesses the structural model (Anderson and Gerbing, 1988).

### 4.1 Measurement model

Measurement models measure the relation between the latent variables and their indicators for both reflective and formative constructs. In this study, all constructs are reflective except computer use, which is formative.

The internal consistency, convergent validity and discriminatory validity must be verified to assess the reflective measurement model. The composite reliability (CR), shown in Appendix B, is higher than 0.7 in all constructs, reflecting internal consistency (Mcintosh et al., 2014). Also, by analysing the loadings of the items, which are all higher than 0,6, we can conclude there is indicator reliability. To demonstrate convergent validity, we verify the average variance extracted (AVE) values of constructs, and they are all higher than 0.5 (please see Appendix B), confirming there is convergent validity (Sarstedt et al., 2017). To analyse discriminant validity, we implemented three methods - the Fornell-Larcker criterion, the loadings and cross-loadings analysis, and the heterotrait-monotrait ratio (HTMT) methodology. The *Fornell-Larcker* criterion supports that the AVE square root of each construct should be higher than the correlation between constructs (Fornell and Larcker, 1981), which Appendix B can confirm. The second criteria support that the loadings should be higher than the respective cross-loadings (Joseph F Hair et al., 2014), which is observed in AppendixC. The HTMT method sustains that the HTMT values should be lower than 0.9 (Joseph F Hair et al., 2017; Sarstedt et al., 2017), confirmed by Appendix D. Thus, all the constructs have discriminant validity.

In order to assess the validity of the formative construct computer use, we assessed the model for multicollinearity using (variance inflation factor) VIF. Table 1 shows the VIF values are all under 5 (Joseph F Hair et al., 2017), as the threshold indicates it should be, so the model does not have multicollinearity problems. In terms of significance, the three items are statistically significant (p < 0.05), as Table 1 confirms, concluding that the formative construct is reliable.

**Table 1:** Formative measurement model evaluation.

Items	VIF	Weights
CU1	1.257	0.220∗
CU2	1.016	0.724∗∗∗
CU3	1.273	0.477∗

Note: ∗p < 0.05, ∗∗p < 0.01, ∗∗∗p < 0.001.

We can conclude that both reflective and formative constructs present a good measurement model. For this reason, we can move to the structural model.

### 4.2 Structural model

**Figure 2:**
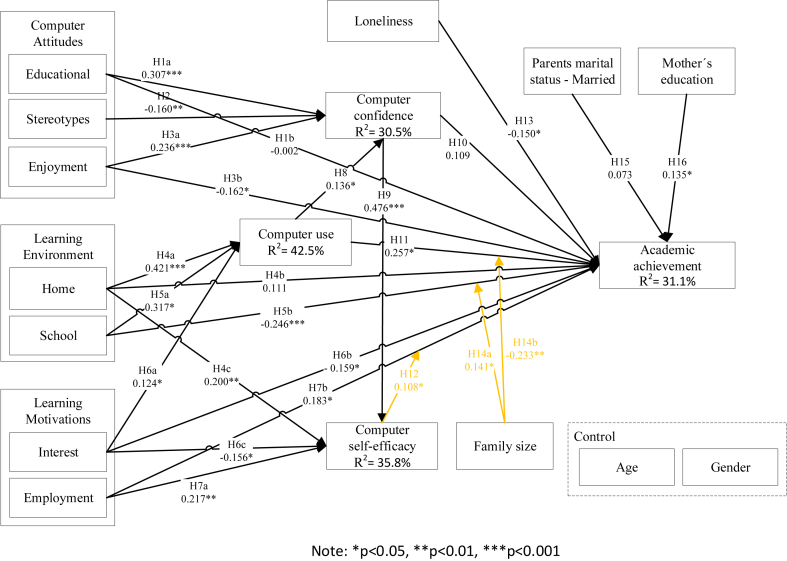
Conceptual model results.

To estimate the structural model, first, we assessed the VIF to check the model for multicollinearity issues. The VIF values are below the threshold of 5 (Sarstedt et al., 2017), so the model does not have multicollinearity problems. To evaluate the statistical significance of the path coefficients, we did a bootstrap with 5000 resamples. Results from the model are presented in Figure 2.

The model explains 30.5% of computer confidence. Educational attitudes (β = 0.307, p < 0.001), stereotype attitudes (β = - 0.160, p < 0.01), enjoyment attitudes (β = 0.236, p < 0.001) and computer use (β = 0.136, p < 0.05) are statistically significant in explaining computer confidence, confirming hypotheses H1a, H2, H3a and H8. The explained variation of computer use is 42,5%. The results show that home environment (β = 0.421, p < 0.001), school environment (β = 0.317, p < 0.05) and interest motivations (β = 0.124, p < 0.05) are statistically significant and have a positive influence on computer use, thus hypotheses H4a, H5a and H6a are supported. The model explains 35.8% of computer self-efficacy. The home environment construct (β = 0.200, p < 0.01), interest motivations (β = - 0.156, p < 0.05), and employment motivations (β = 0.217, p < 0.01) are statistically significant however, home environment and employment motivation show a positive influence on computer self-efficacy, supporting hypotheses H4c, H7a and interest motivations show a negative influence on computer self-efficacy where we expected a positive influence, rejecting H6c.

The model explains 31.1% of students' academic achievement. Enjoyment attitudes (β = - 0.162, p < 0.05), employment motivations (β = 0.183, p < 0.05), computer use (β = 0.257, p < 0.05), loneliness (β = - 0.150, p < 0.05) and mother's education (β = 0.135, p < 0.05) are statistically significant in explaining academic achievement, supporting the hypotheses, H3b, H7b, H11, H13 and H16. We reject respective hypotheses H5b and H6b respectively, despite school environment (β = - 0.246, p < 0.001) and interest motivations (β = - 0.159, p < 0.05), being statistically significant, because we suggested that school environment and interest motivations would positively influence academic achievement, and the results observe a negative influence. Educational attitudes (β = -0.003, p > 0.05), home environment (β = 0.100, p > 0.05), computer confidence (0.105, p > 0.05) and parental marital status (β = 0.067, p > 0.05) show a non-significant effect on explaining academic achievement, rejecting H1b, H4b, H10 and H15. The moderation effect of computer self-efficacy in employment motivations (β = 0.108, p < 0.05) is statistically significant, supporting H12. The moderation effect of family size on school environment (β = 0.141, p < 0.05) and on computer use (β = - 0.233, p < 0.01) is statistically significant, supporting H14a and H14b.

Table 2 summarises the research hypotheses results. We can conclude that 17 of the 25 proposed hypotheses were supported.

**Table 2:** Research hypotheses results.

	Independent variable		Dependent variable	Moderator	$\hat{\beta }$	Findings	Conclusion
H1a	Educational attitudes (EdA)	→	Computer confidence (CC)	n.a.	0.307	∗∗∗	Supported
H1b	Educational attitudes (EdA)	→	Academic achievement (AA)	n.a.	-0.002	Non-significant	Not supported
H2	Stereotype attitudes (SA)	→	Computer confidence (CC)	n.a.	-0.160	∗∗	Supported
H3a	Enjoyment attitudes (EjA)	→	Computer confidence (CC)	n.a.	0.236	∗∗∗	Supported
H3b	Enjoyment attitudes (EjA)	→	Academic achievement (AA)	n.a.	-0.162	∗	Not supported
H4a	Home environment (HE)	→	Computer use (CU)	n.a.	0.421	∗∗∗	Supported
H4b	Home environment (HE)	→	Academic achievement (AA)	n.a.	0.111	Non-significant	Not supported
H4c	Home environment (HE)	→	Computer self-efficacy (CS)	n.a.	0.200	∗∗	Supported
H5a	School environment (SE)	→	Computer use (CU)	n.a.	0.317	∗	Supported
H5b	School environment (SE)	→	Academic achievement (AA)	n.a	-0.246	∗∗∗	Not supported
H6a	Interest motivations (IM)	→	Computer use (CU)	n.a.	0.124	∗	Supported
H6b	Interest motivations (IM)	→	Academic achievement (AA)	n.a.	-0.159	∗	Not supported
H6c	Interest motivations (IM)	→	Computer self-efficacy (CS)	n.a.	-0.156	∗	Not Supported
H7a	Employment motivations (EM)	→	Computer self-efficacy (CS)	n.a.	0.217	∗∗	Supported
H7b	Employment motivations (EM)	→	Academic achievement (AA)	n.a	0.183	∗	Supported
H8	Computer use (CU)	→	Computer confidence (CC)	n.a.	0.136	∗	Supported
H9	Computer confidence (CC)	→	Computer self-efficacy (CS)	n.a.	0.476	∗∗∗	Supported
H10	Computer confidence (CC)	→	Academic achievement (AA)	n.a.	0.109	Non-significant	Not supported
H11	Computer use (CU)	→	Academic achievement (AA)	n.a	0.257	∗	Supported
H12	Employment Motivations ∗ Computer self-efficacy	→	Academic achievement (AA)	Computer Self-efficacy	0.108	∗	Supported
H13	Loneliness (L)	→	Academic achievement (AA)	n.a.	-0.150	∗	Supported
H14a	School Environment ∗ Family size	→	Academic achievement (AA)	Family size	0.141	∗∗	Supported
H14b	Computer Use ∗ Family size	→	Academic achievement (AA)	Family size	-0.233	∗∗	Supported
H15	Parental marital status (MS)	→	Academic achievement (AA)	n.a.	0.073	Non-significant	Not supported
H16	Mother's education (ME)	→	Academic achievement (AA)	n.a	0.135	∗	Supported

**Notes:** n.a. - not applicable; ∗ significant at p < 0.05; ∗∗ significant at p < 0.01; ∗∗∗ significant at p < 0.001.

## 5 Discussion

This research model contributes to and extends the literature review on computers and academic achievement. This study relates academic achievement with loneliness, family and computer-related variables such as computer confidence, computer self-efficacy, computer attitudes, computer learning motivations and computer learning environments.

The results show that educational and enjoyment computer attitudes positively influence computer confidence, while stereotype attitudes negatively influence it. We expected this negative relation regarding stereotypes since there are the same results regarding stereotypes on gender and age (Punter et al., 2017), although similar results concerning stereotypes on computer users have not yet been found. As for the influence of attitudes on academic achievement, educational computer attitudes do not have a statistically significant relationship with academic achievement. On the other hand, enjoyable computer attitudes have a significant negative impact on academic achievement, which leads us to conclude that there is no relation between computers as an educational tool and academic achievement. In fact, apart from some specific high school vocational courses oriented to computing skills, most classes happen in a classic lecture setting and rely mostly on textbook manuals as learning tools, which can help explain the results regarding educational computer attitude. However, using computers for recreational purposes negatively influences students' academic achievement, as similar results have already been observed - students who play more video games have a lower achievement (Tang and Patrick, 2018). Two possible reasons can explain this phenomenon. First, because young adults are so engaged and skilled with technology use for game playing and social media that they do not make the best use of these skills for academic purposes, for instance (Gurung and Rutledge, 2014) and second, because excessive use and multitasking can lead to distractions and lack of time to study (Rashid and Asghar, 2016).

The construct computer use, measured as the frequency of use, positively impacts computer confidence and academic achievement. Thus, the greater the use of computers, the more confident students are while using them, and so the more use of the computer, the better the performance achieved. Several other studies contradict the negative influence verified between school environment and academic achievement (Bayrak and Bayram, 2010; Carle et al., 2009; Murillo-Zamorano et al., 2019). However, this can be explained by the rapid development of computer technology and the massive use of computers at home compared to the lack of use at school due to schools' technology being obsolete, and students preferring the home environment.

The results demonstrate that computer use works as a full mediator for home environment and academic achievement since there is no relation between home environment and academic achievement, contrary to another study (Fairlie et al., 2010). However, with computer use as a mediator, we suggest that the home environment influences academic achievement when computer use increases since there is a positive relation between home environment and computer use (Hsu and Huang, 2006), i.e., students who use a computer at home have better results. Also, computer use works as a partial mediator for the school environment and academic achievement. Hence, we suggest that, although the use of computers at school already directly (but negatively) influences students' performance, computer use mediates this relation positively. This effect is likely due to the fact that even though there is an effort to implement digital transformation in the education sector, there is still a lack of computers at schools: most students do not have easy access to computers in school (high schools in Portugal have an average 4.2 students per computer), but those who use them benefit on their grades. These results allow us to confirm our second contribution, the investigation of the mediation effect of computer use between the factors identified and academic achievement. The mediation results are shown in Table 3.

**Table 3:** Hypotheses testing on mediation.

Effect of	Indirect effect (a x b) (t-value)	Direct effect (c) (t-value)	Sign (a x b x c)	Interpretation	Conclusion
HE - > CU - > AA	0.117∗ (2.025)	0.111 (1.560)	+	Full mediation	H4c supported
SE - > CU - > AA	0.086∗ (2.271)	-0.246 ∗∗∗ (3.958)	+	Complementary mediation	H4c supported

Note: ∗ |t|> 1.96 and p-value = 0.05.; ∗∗ |t| > 2.57 and p-value = 0.01; ∗∗∗ |t| > 3.291 and p-value = 0.001.

Regarding motivations, interest motivation impacts computer use positively, as concluded by other similar findings (Rohatgi et al., 2016), i.e. the more interested students are in computers, the more they use them. Nonetheless, it negatively influences academic achievement and computer self-efficacy, concluding that the bigger the interest motivation, the more the use of computers but the lower the achievement and the computer self-efficacy. These two negative relations are quite controversial compared to the literature. However, it may mean that the more interest in computers, the more use for recreational purposes, negatively impacting academic achievement (Rashid and Asghar, 2016). The more interest students have in computers, the more knowledge of using the devices, and the perceived efficacy starts to decrease. Thus further research is needed to draw any conclusions on this.

Computer confidence has a strong positive effect on computer self-efficacy, meaning that the perceived computer self-efficacy increases when the confidence in the device is higher, as stated in similar findings (Hatlevik and Bjarnø, 2021). Although, we cannot conclude there is a relation between computer confidence and academic achievement. All the previous results allow us to reflect on the influence that the computer-related variables studied have on the student performance, contributing with data for future research and confirming our first contribution of the study.

The loneliness construct, used as a measure of coronavirus effects, negatively influenced academic achievement, as expected. While students were in lockdown having remote classes, without any presential contact with their school, teachers, and colleagues, the feeling of loneliness and isolation negatively impacted their performance indeed, as observed in our results. These results confirm our contribution to understanding how the COVID-19 pandemic influences students’ academic achievement. Recent studies found negative impacts of loneliness (Roy et al., 2020) on students, demonstrating the importance of cooperating with colleagues (Torres-Díaz et al., 2016). However, there are yet no results of the direct impact of loneliness deriving from the pandemic on academic achievement.

**Figure 3:**
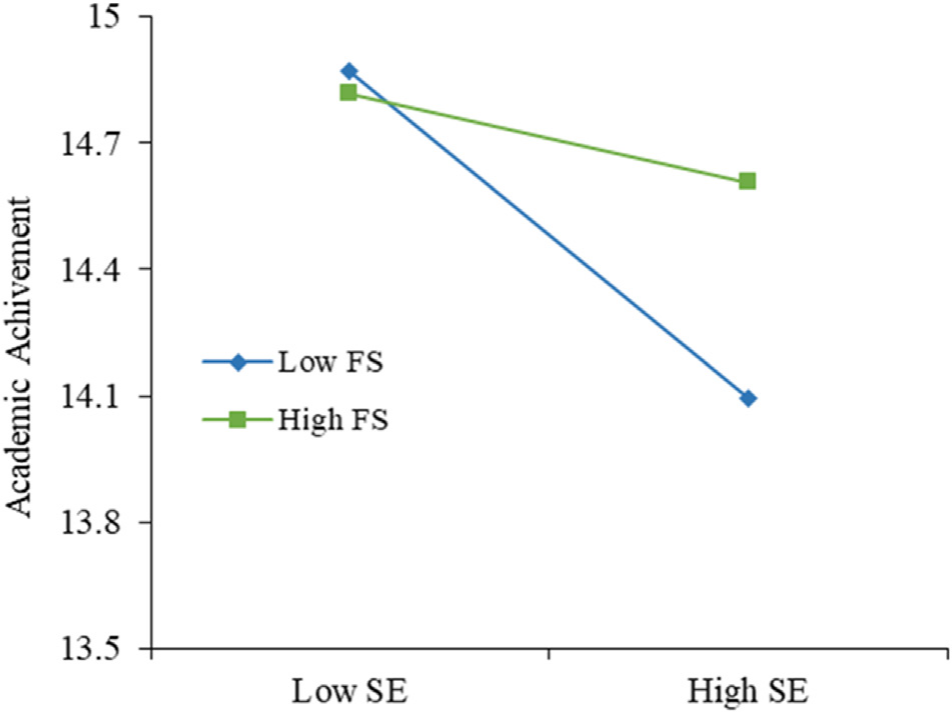
Structural model (variance-based technique) for academic achievement.

**Figure 4:**
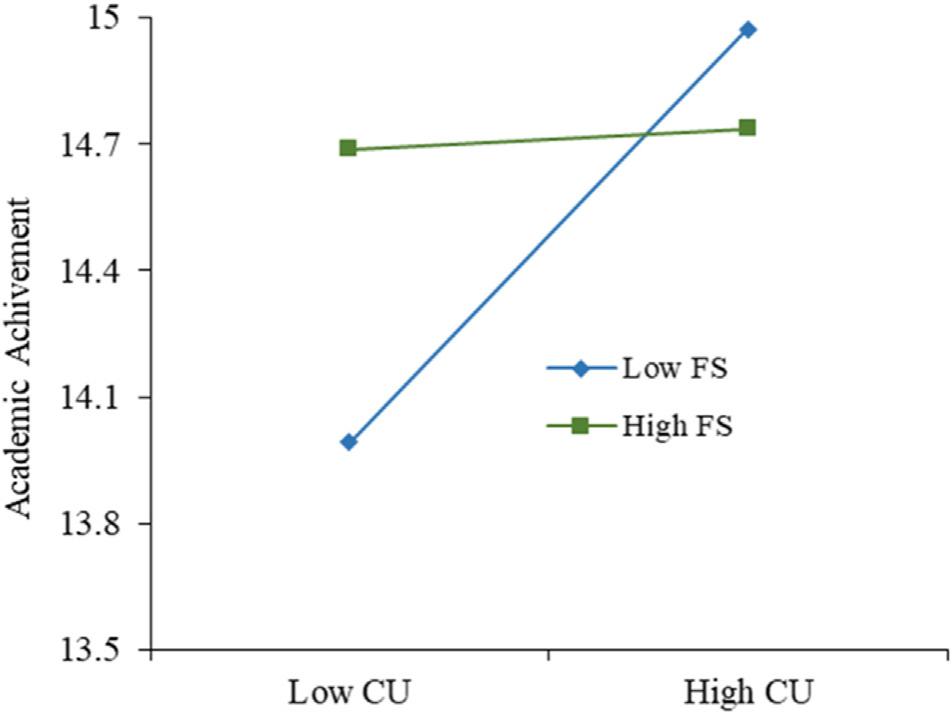
Structural model (variance-based technique) for academic achievement.

**Figure 5:**
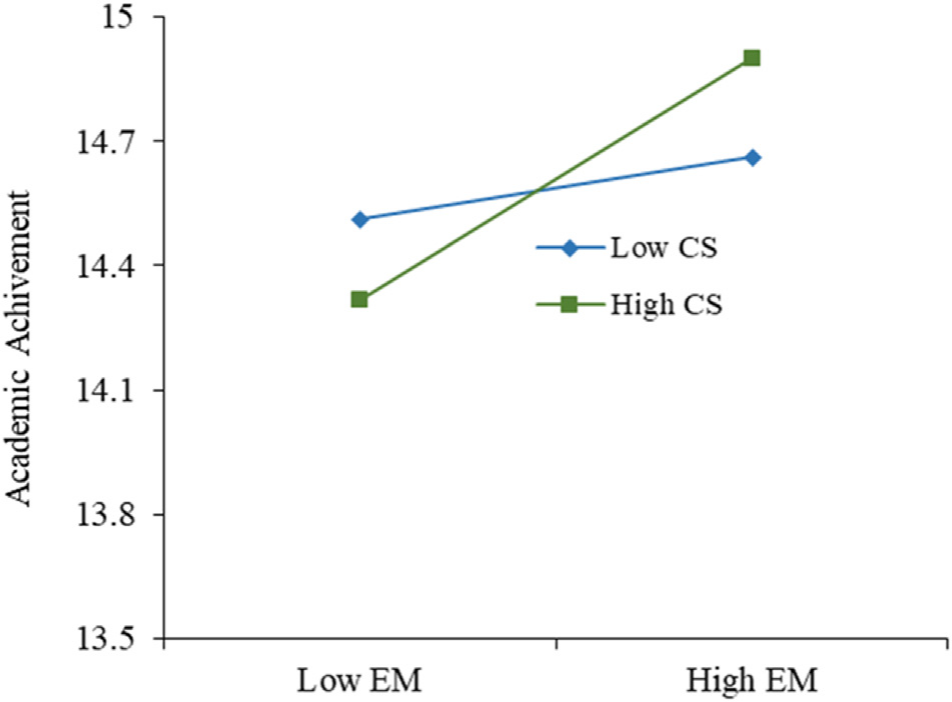
Structural model (variance-based technique) for academic achievement.

There are three moderation hypotheses using family size and computer self-efficacy. From the family size moderator, we can conclude that family size influences the relation between school environment and academic achievement. In Figure 3, we can see that when the family size decreases, the negative impact the school environment has on academic achievement increases, suggesting that the smaller the family, the students tend to have worse grades when studying in a school environment. Regarding family size in the relation between computer use and academic achievement, shown in Figure 4, when the family size decreases, computer use is more important to explain academic achievement because when the family is small, students need to use the computer more to achieve better results. Relating to the computer self-efficacy moderator, in Figure 5, it impacts the relationship between employment motivations and academic achievement positively, meaning that the better students perceive their computer self-efficacy, the stronger positive impact employment motivation has on academic achievement. This effect can be explained due to the increase of technological jobs: students who feel more capable in their computer skills (with a higher computer self-efficacy) and are more motivated to pursue a technological career have higher academic achievement. These results allow us to confirm our second contribution, the investigation of the moderation effect family size and computer self-efficacy.

In this study, we found that marital status does not have any effect on academic achievement, but mothers' education has a positive impact on students' achievement, reinforcing the literature (Abosede and Akintola, 2016).

### 5.1 Practical implications

Academic achievement is a widely topic studied because there is an ongoing concern for understanding the factors that lead to better academic achievements. Since students practically depend on computers for school nowadays, we tried to relate the most studied computer variables in the literature with academic achievement, expecting results that answer the gaps identified in the literature. To our knowledge, no study has yet provided a conclusion on the influence of loneliness provoked by the COVID-19 pandemic on academic achievement, neither of interest and employment motivations on AA. Moreover, there is no consensus in the literature on the influence of the use of computers for fun and academic performance. We can contribute to the literature with the answers to these questions: students who feel lonely have worse academic achievement, students motivated by an interest in computers have worse academic achievement and students motivated by the expectation of having a good job have better grades. Also, enjoyable computer attitudes negatively influence academic achievement, so the students who find the computer a good tool for recreational purposes have worse grades.

Contrary to the literature, we found that computer confidence does not influence academic achievement; apart from this, we concur with the available results published by other researchers. There are clear positive implications on using computers in education, and consequently, in students' outcomes. Therefore, teachers and parents should encourage using computers in adolescents' education to improve their school performance and future.

### 5.2 Limitations and further research

The present study has some limitations that point to future research directions on the role of students' academic achievement and its predictors. First, the data collected does not have sufficient diversity in country dispersity and gender balance since most participants were girls hailing from Portugal. Also, better results can be obtained with a more significant sample. Secondly, the fact that we are going through a pandemic forced schools and students to attend classes online, which on the one hand, is an advantage because it provides the opportunity to study loneliness deriving from the pandemic. On the other hand, it could bias the students' answers to the questionnaire and the subsequent results because their opinion on computers could have changed during home-schooling compared to the usual previous schooling method since the literature is related to regular presential school attendance.

In further research, other factors regarding loneliness should be studied to understand the impact of coronavirus on students' lives better, comparing pre-pandemic and pandemic daily computer usage. Other factors such as addiction to technology should be analysed.

## 6 Conclusions

This study proposes a theoretical model on the influence of several computer factors on the academic achievement of high school students. The results, in general, empirically support the literature in similar findings. The proposed conceptual model explains 31.1% of academic achievement. We found that students who use computers for recreational purposes or feel that a computer is a tool to "pass the time" or play games are those who have the worst grades. We can conclude this through the negative relation between enjoyment attitudes and academic achievement. Nevertheless, there is no relation between students who perceive computers as an educational tool and their academic achievement. We believe this conclusion results from how teenagers use their computers and smartphones excessively, not prioritising the use for school, leading to the observed results. Our results also show that there are still stereotypes about who uses computers most. Respondents believe that peers who play sports do not have the same likelihood of using computers excessively, and those that frequently use computers are not sociable. This mindset leads to less confidence in computers.

A significant conclusion was found regarding the computer use environment, though the mediation effect of computer use. When students use the computer at home, they need to use it frequently to influence their academic achievement, but when students use the computer at school, it will influence their academic achievement positively independently of the frequency of use. However, the frequency of computer use itself influences academic achievement. As we expected, the feelings of loneliness associated with the coronavirus negatively influence students' academic achievement, an important new conclusion in the literature. The moderation effect on family size allows us to conclude that students with a smaller family tend to have worse grades when studying in a school environment and need to use computers more to have better school results than those in larger families. Moreover, the moderation effect on computer self-efficacy lets us conclude that students who perceive better computer self-efficacy, have better grades and academic achievement is influenced by employment motivation.

## Declarations

### Author contribution statement

Sofia Simões: Conceived and designed the experiments; Performed the experiments; Analyzed and interpreted the data; Contributed reagents, materials, analysis tools or data; Wrote the paper.

Tiago Oliveira: Conceived and designed the experiments; Analyzed and interpreted the data; Contributed reagents, materials, analysis tools or data; Wrote the paper.

Catarina Nunes Analyzed and interpreted the data; Wrote the paper.

### Funding statement

This work was supported by 10.13039/501100001871FCT (Fundação para a Ciência e a Tecnologia) under project DSAIPA/DS/0032/2018 (DS4AA).

### Data availability statement

Data will be made available on request.

### Declaration of interests statement

The authors declare no conflict of interest.

### Additional information

No additional information is available for this paper.

## Figures and Tables

**Table:** 

Constructs	Items	Author
Educational attitudes ^1^	EdA1 – Computers are fascinating	(Levine and Donitsa-Schmidt, 1998)
	EdA2 – A computer is an educational tool	
	EdA3 – A computer is an effective learning tool	
	EdA4 – One can learn new things from a computer	
	EdA5 – You can learn a lot from using a computer	
Stereotypes attitudes ^2^	SA1 - People who like computers are often not very sociable	(Levine and Donitsa-Schmidt, 1998)
	SA2 – People who like computers are usually weird	
	SA3 – I would not expect a good athlete to like computers	
	SA4 – People who like computers are often squares	
Enjoyment attitudes ^3^	EjA1 – Working with a computer is a good way to pass the time	(Levine and Donitsa-Schmidt, 1998)
	EjA2 – I prefer computer games to other games	
	EdA3 – The computer stops me from getting bored	
	EdA4 – I use the computer when I have nothing else to do	
Home environment ^4^	HE1 – I work with a computer at home most of the time	(Hsu and Huang, 2006)
	HE2 – When I am at home, I am always using a computer	
School environment ^5^	SE1 – Most of my teachers encourage me to learn with computers	
	SE2 – The computer learning facilities at my school are good	(Hsu and Huang, 2006)
	SE3 – I use computers at school a lot	
Interest motivations ^6^	IM1 – I enjoy using computers	(Hsu and Huang, 2006)
	IM2 – I would take any opportunity to use computers	
	IM3 – I am motivated when I use a computer	
Employment motivations ^7^	EM1 – Computer skills will be helpful for me to get a good job	(Hsu and Huang, 2006)
	EM2 – I will need adequate computer skills for my future work	
	EM3 – Computer skills will improve my curriculum	
	EM4 – I will need a computer to work in my daily job	
Computer use ^8^	CU1 – The extent of computer use at school	(Hsu and Huang, 2006)
	CU2 – The frequency of general computer use at home	
	CU3 – The frequency of general computer use in school	
Computer confidence ^9^	CC1 – I feel comfortable working with computers	(Levine and Donitsa-Schmidt, 1998)
	CC2 – I find using a computer easy	
	CC3 – I learn more rapidly when I use a computer	
Computer self-efficacy ^10^	CS1 – I can skillfully use a computer to make a report/write an essay.	(Hsu and Huang, 2006)
	CS2 – I can skillfully use a computer to analyse numerical data.	
	CS3 – I can easily write a simple program for a computer.	
	CS4 – I can skillfully use a computer to organise information.	
Loneliness ^11^	L1 – How often do you feel that you lack companionship?	(Liu et al., 2020)
	L2 – How often do you feel left out?	
	L3 – How often do you feel isolated from others?	
Academic achievement ^12^	AA1 – Mathematical achievement	(Gonzalez-pienda et al., 2002)
	AA2 – Verbal achievement	
	AA3 – Remaining subjects	
	AA4 – Global achievement in remaining areas.	
Family size ^13^	FS1: What is your family size?	(Tesfagiorgis et al., 2020)
Parents Marital Status ^14^	MS1: What is your parent's marital status?	(Abosede and Akintola, 2016)
Mothers' Education ^15^	PE1: What is the highest educational level your mother completed	(Abosede and Akintola, 2016)
Age ^16^	A1: Age	(Chowa et al., 2015)
Gender ^17^	G1: Gender	(Chowa et al., 2015)

Notes: ^1, 2, 3, 4, 5, 6, 7, 9, 10^ Range scale from 1 (Strongly Disagree) to 5 (Strongly Agree); ^8^ Range scale from 1 (Never) to 5 (Everyday); ^11^ Ordinal Scale (Hardly ever, some of the time, often); ^12^ Ratio scale from 0 to 20 (number); ^13^ Nominal scale (number); ^14^ Nominal scale (married, divorced, in a domestic partnership, widowed, other); ^15^ Ordinal scale (less than high school, high school or equivalent, bachelor's degree, master's degree, doctorate, other); ^16^ Ratio scale (number); ^17^ Nominal scale (male, female).

**Table:** 

	Mean	SD	CR	EdA	SA	EjA	HE	SE	IM	EM	CU	CC	CS	L	FS	MS	ME	AA
Educational attitudes (EdA)	4.345	0.609	0.880	**0.772**														
Stereotypes attitudes (SA)	1.533	0.711	0.881	-0.312	**0.807**													
Enjoyment attitudes (EjA)	3.425	0.941	0.849	0.307	0.023	**0.765**												
Home environment (HE)	3.325	0.995	0.847	0.383	-0.054	0.313	**0.858**											
School environment (SE)	2.559	0.888	0.780	0.176	0.001	0.042	0.246	**0.736**										
Interest motivations (IM)	3.837	0.814	0.845	0.481	-0.125	0.473	0.466	0.233	**0.804**									
Employment motivations (EM)	4.230	0.716	0.854	0.473	-0.145	0.142	0.360	0.227	0.292	**0.772**								
Computer use (CU)	3.557	0.799		0.284	-0.065	0.170	0.557	0.449	0.394	0.353								
Computer confidence (CC)	4.113	0.755	0.865	0.468	-0.259	0.349	0.291	0.187	0.494	0.268	0.274	**0.826**						
Computer self-efficacy (CS)	3.930	0.779	0.846	0.353	-0.259	0.173	0.344	0.151	0.235	0.371	0.279	0.516	**0.761**					
Loneliness (L)	2.596	1.119	0.920	-0.081	0.142	0.155	0.025	-0.055	-0.010	-0.041	-0.093	-0.096	-0.132	**0.891**				
Family size (FS)	3.811	1.066	1.000	-0.104	0.065	0.010	0.079	-0.005	-0.042	0.003	-0.009	0.001	0.002	0.014	**1.000**			
Marital status (MS)	1.000	0.000	1.000	-0.078	0.072	-0.042	0.027	-0.100	-0.052	0.059	-0.002	0.016	0.003	-0.057	0.152	**1.000**		
Mother education (ME)	13.291	4.006	1.000	0.087	-0.009	-0.061	0.002	-0.091	-0.076	0.107	-0.034	0.006	0.131	-0.117	0.025	0.070	**1.000**	
Academic achievement (AA)	14.597	2.347	0.921	0.043	-0.092	-0.147	0.170	-0.102	-0.086	0.203	0.190	0.053	0.135	-0.205	0.086	0.194	0.191	**0.864**

Note: Values in diagonal (bold) are the AVE square root.

**Table:** 

	CC	CS	EjA	HE	SE	EdA	SA	L	EM	IM	AA
CC3	**0.871**	0.466	0.279	0.240	0.163	0.430	-0.253	-0.082	0.296	0.453	0.079
CC4	**0.885**	0.505	0.273	0.190	0.131	0.344	-0.248	-0.147	0.237	0.354	0.110
CC5	**0.713**	0.280	0.331	0.315	0.177	0.394	-0.123	0.010	0.107	0.429	-0.088
CS1	0.367	**0.730**	0.098	0.253	0.085	0.320	-0.204	-0.115	0.305	0.110	0.166
CS2	0.324	**0.777**	0.089	0.184	0.067	0.219	-0.133	-0.083	0.282	0.149	0.052
CS3	0.444	**0.703**	0.186	0.293	0.205	0.208	-0.158	-0.097	0.220	0.188	0.057
CS4	0.416	**0.829**	0.142	0.298	0.092	0.314	-0.277	-0.101	0.317	0.260	0.125
EjA1	0.337	0.175	**0.802**	0.231	0.072	0.315	-0.048	0.137	0.193	0.453	-0.176
EjA2	0.240	0.118	**0.713**	0.259	-0.035	0.199	0.085	0.023	0.075	0.322	-0.116
EjA3	0.228	0.065	**0.839**	0.210	0.033	0.209	0.034	0.171	0.073	0.352	-0.113
EjA4	0.231	0.158	**0.699**	0.272	0.047	0.175	0.033	0.148	0.045	0.271	0.003
HE3	0.241	0.353	0.142	**0.901**	0.211	0.371	-0.125	-0.009	0.392	0.361	0.229
HE4	0.266	0.221	0.443	**0.812**	0.214	0.275	0.060	0.062	0.202	0.457	0.037
SE1	0.143	0.098	0.034	0.268	**0.752**	0.228	-0.035	-0.001	0.286	0.235	-0.050
SE2	0.124	0.195	0.003	0.166	**0.683**	0.158	-0.068	-0.083	0.145	0.060	-0.016
SE3	0.144	0.056	0.051	0.104	**0.770**	0.004	0.095	-0.048	0.063	0.197	-0.151
EdA1	0.436	0.274	0.478	0.325	0.110	**0.701**	-0.180	0.016	0.339	0.530	-0.102
EdA2	0.380	0.304	0.126	0.258	0.147	**0.836**	-0.219	-0.093	0.382	0.312	0.095
EdA3	0.348	0.251	0.155	0.307	0.199	**0.791**	-0.258	-0.144	0.321	0.357	0.050
EdA4	0.289	0.274	0.146	0.310	0.083	**0.754**	-0.316	-0.077	0.392	0.268	0.119
EdA5	0.314	0.252	0.220	0.268	0.135	**0.771**	-0.256	-0.026	0.396	0.337	0.039
SA2	-0.229	-0.221	0.020	-0.007	0.007	-0.206	**0.821**	0.139	-0.055	-0.066	-0.041
SA3	-0.263	-0.209	-0.023	-0.089	-0.029	-0.370	**0.887**	0.110	-0.200	-0.197	-0.095
SA4	-0.096	-0.212	0.168	-0.017	0.010	-0.116	**0.680**	0.105	-0.035	0.061	-0.106
SA5	-0.189	-0.214	0.002	-0.041	0.031	-0.239	**0.827**	0.108	-0.131	-0.103	-0.076
L1	-0.049	-0.125	0.130	-0.002	-0.052	-0.035	0.102	**0.872**	0.004	-0.010	-0.196
L2	-0.143	-0.112	0.169	0.014	-0.042	-0.091	0.148	**0.888**	-0.078	-0.035	-0.162
L3	-0.075	-0.114	0.120	0.054	-0.052	-0.094	0.134	**0.913**	-0.043	0.015	-0.186
EM1	0.261	0.262	0.215	0.262	0.199	0.418	-0.066	-0.065	**0.719**	0.309	0.123
EM2	0.201	0.307	0.071	0.297	0.179	0.361	-0.162	0.012	**0.836**	0.227	0.178
EM3	0.190	0.337	0.052	0.238	0.220	0.370	-0.087	-0.025	**0.801**	0.144	0.160
EM4	0.186	0.223	0.129	0.331	0.089	0.317	-0.134	-0.062	**0.723**	0.253	0.163
IM1	0.452	0.291	0.405	0.428	0.141	0.459	-0.218	-0.095	0.287	**0.860**	-0.001
IM2	0.374	0.122	0.356	0.341	0.231	0.278	0.020	0.095	0.215	**0.816**	-0.144
IM4	0.350	0.108	0.384	0.342	0.219	0.418	-0.056	0.012	0.182	**0.731**	-0.095
AA1	-0.021	0.062	-0.124	0.078	-0.151	-0.008	-0.097	-0.103	0.106	-0.126	**0.751**
AA2	0.054	0.176	-0.138	0.141	-0.148	0.068	-0.050	-0.183	0.177	-0.087	**0.891**
AA3	0.080	0.096	-0.117	0.170	-0.024	0.023	-0.043	-0.182	0.192	-0.038	**0.856**
AA4	0.062	0.124	-0.131	0.188	-0.043	0.056	-0.123	-0.226	0.216	-0.055	**0.947**

**Table:** 

Constructs	EdA	SA	EjA	HE	SE	IM	EM	CC	CS	L	FS	MS	ME	AA
Educational attitudes (EdA)														
Stereotypes attitudes (SA)	0.354													
Enjoyment attitudes (EjA)	0.347	0.122												
Home environment (HE)	0.508	0.158	0.489											
School environment (SE)	0.277	0.139	0.088	0.399										
Interest motivations (IM)	0.592	0.202	0.605	0.681	0.360									
Employment motivations (EM)	0.594	0.168	0.180	0.496	0.331	0.387								
Computer confidence (CC)	0.580	0.294	0.450	0.434	0.285	0.658	0.340							
Computer self-efficacy (CS)	0.437	0.326	0.216	0.469	0.272	0.285	0.477	0.657						
Loneliness (L)	0.114	0.169	0.207	0.066	0.091	0.109	0.074	0.131	0.160					
Family size (FS)	0.109	0.086	0.043	0.096	0.081	0.043	0.075	0.028	0.011	0.015				
Maritus Status (MS)	0.079	0.067	0.070	0.031	0.130	0.079	0.065	0.039	0.023	0.061	0.152			
Mothers education (ME)	0.105	0.039	0.091	0.035	0.162	0.095	0.118	0.034	0.150	0.123	0.025	0.070		
Academic Achievement (AA)	0.121	0.113	0.177	0.202	0.163	0.144	0.242	0.145	0.158	0.228	0.091	0.209	0.202	
